# Highly Sensing and Selective Performance Based on Bi-Doped Porous ZnSnO_3_ Nanospheres for Detection of *n*-Butanol

**DOI:** 10.3390/s22176571

**Published:** 2022-08-31

**Authors:** Lili Jiang, Qi Cui, Ruijia Zhang, Wenqiang Zhang

**Affiliations:** School of Materials Science and Engineering, Lanzhou University of Technology, Langongping Road, Lanzhou 730050, China

**Keywords:** gas sensor, ZnSnO_3_, *n*-butanol, hollow sphere

## Abstract

In this study, pure zinc stannate (ZnSnO_3_) and bismuth (Bi)-doped ZnSnO_3_ composites (Bi-ZnSnO_3_) were synthesized via the in situ precipitation method, and their microstructures, morphologies, chemical components, sizes, and specific surface areas were characterized, followed by testing their gas sensing properties. The results revealed that Bi-ZnSnO_3_ showed superior gas sensing properties to *n*-butanol gas, with an optimal operating temperature of 300 °C, which was 50 °C lower than that of pure ZnSnO_3_. At this temperature, moreover, the sensitivity of Bi-ZnSnO_3_ to *n*-butanol gas at the concentration of 100 ppm reached as high as 1450.65, which was 35.57 times that (41.01) of ammonia gas, 2.93 times that (495.09) of acetone gas, 6.02 times that (241.05) of methanol gas, 2.54 times that (571.48) of formaldehyde gas, and 2.98 times that (486.58) of ethanol gas. Bi-ZnSnO_3_ had a highly repeatable performance. The total proportion of oxygen vacancies and chemi-adsorbed oxygen in Bi-ZnSnO_3_ (4 wt%) was 27.72% to 32.68% higher than that of pure ZnSnO_3_. Therefore, Bi-ZnSnO_3_ has considerable potential in detecting *n*-butanol gas by virtue of its excellent gas-sensing properties.

## 1. Introduction

*n*-Butanol, a typical volatile organic compound, has been widely applied to the production of various paint solvents, extractants, and plastic rubber products [1,2]. *n*-Butanol, which is flammable, gives off a unique pungent smell and tends to form explosive mixtures with air [3]. High-concentration *n*-butanol gas is hazardous to the human body, causing symptoms such as dizziness, headache, drowsiness, and dermatitis if exposed at >50 ppm for a long time [4,5]. Therefore, *n*-butanol gas must be rapidly and effectively monitored for the sake of human health and safety, but expensive and cumbersome monitoring instruments are usually required for traditional *n*-butanol detection methods, which are also marred by their complex operation, long response time(T(res)), etc. [6,7]. Hence, it is worthwhile to develop highly sensitive, highly selective gas sensors that can effectively monitor *n*-butanol. A variety of gas sensors have been developed, including semiconductor metal oxide gas sensors, primary battery-type oxygen sensors, and catalytic combustion-type sensors [8].

Among semiconductor metal oxides, the most extensively applied gas-sensitive materials at present, n-type semiconductors are the most studied, such as SnO_2_, TiO_2_, Fe_2_O_3_, In_2_O_3_, LaFeO_3_, WO_3_, and ZnO, and the gas-sensitive devices made of such materials integrate the merits of low cost, strong stability, easy manufacturing, and convenient use [9,10,11]. Nevertheless, single-metal-oxide gas sensors can no longer meet operational needs due to their high operating temperature and low sensitivity [12]. As a typical n-type metal oxide semiconductor, zinc stannate (ZnSnO_3_), which can be easily prepared and conveniently used at a low cost, has been used to prepare sensor elements to detect all kinds of volatile organic gases [13]. The gas-sensing properties of ZnSnO_3_ with different morphologies have been explored, including ZnSnO_3_ cubes, porous ZnSnO_3_ nanospheres, and ZnSnO_3_ nanorods [14]. Zeng et al. [15] successfully synthesized ZnSnO_3_ nanotubes through the hydrothermal method; Mahmood et al. [16] prepared NiO/ZnSnO_3_ composites using the electrospinning technique; Zhou et al. [17] prepared hollow ZnSnO_3_ through the co-precipitation method. Among these ZnSnO_3_ materials of differing morphology, porous ZnSnO_3_ nanospheres have aroused the most attention, since porous structures feature a large specific surface area and more activated adsorption sites, making them quite conducive to gas diffusion and mass transfer and facilitating oxygen adsorption, qualities that make ZnSnO_3_ strongly gas-responsive. However, pure ZnSnO_3_ gas sensors are prone to the same defects as other semiconductor metal oxide gas sensors, e.g., poor selectivity, weak response, and high operating temperature. Hence, initial efforts have been made to improve the gas-sensing properties of pure ZnSnO_3_, including doping and compounding with other semiconductor materials.

With an ionic radius of 1.03 Å, which differs greatly from that of Sn (0.69 Å) and Zn (0.74 Å), bismuth (Bi) is very prone to cause lattice distortion if doped into the ZnSnO_3_ matrix, leading to many crystal defects and further enhancing the gas-sensing properties of ZnSnO_3_. Mutkule et al. [18] synthesized Bi^3+^-doped spinel cobalt ferrite, a gas-sensitive material; Cai et al. [19] prepared flower-shaped Bi-doped rGO/Co_3_O_4_ nanohybrids; Ma et al. [20] prepared porous Bi-doped SnO_2_ nanosheets via electrospinning. Therefore, it is feasible to enhance the gas-sensing properties of ZnSnO_3_-based gas sensors by doping Bi into ZnSnO_3_.

In this study, pure ZnSnO_3_ and Bi-doped ZnSnO_3_ composites (Bi-ZnSnO_3_) were synthesized via in situ precipitation. Next, the prepared pure ZnSnO_3_ and Bi-ZnSnO_3_ were characterized by X-ray diffractometry (XRD), scanning electron microscopy (SEM), transmission electron microscopy (TEM), a Brunauer–Emmett–Teller (BET) analyzer, and X-ray photoelectron spectroscopy (XPS). Then, the gas sensing properties of the two materials to *n*-butanol were tested, including their optimal operating temperature, sensitivity, T(res)/recovery time T(rec), selectivity, and repeatability.

## 2. Experiments and Methods

### 2.1. Materials and Reagents

The reagents used included absolute ethanol, SnCl_4_•5H_2_O, Bi(NO_3_)_3_•6H_2_O, and NaOH (purchased from Tianjin Chemical Reagent Factory), as well as Zn(NO_3_)_2_•7H_2_O (bought from Junli Chemical Production Factory). All being analytically pure, the chemical reagents were directly used in the experiments without further purification.

### 2.2. Material Characterization

SEM (TESCAN MIRA, Brno, Czech Republic) was done to observe material microstructures, before which the test materials were coated with a thin layer of an electrically conducting material to ensure sufficient conductivity. Material microstructures were also observed via TEM (FEI Talos F200X, FEI, Hillsboro, OR, USA), and the crystal structures of samples were analyzed with XRD (D8 ADVANCE/AXS, Bruker, Saarbrücken, Germany). The specific surface area and the average pore size of the test materials were analyzed with the BET analyzer (ASAP-2460, Micromeritics Instrument Corp., Norcross, GA, USA). Then, the chemical element composition and the compound structure in the test materials were analyzed by XPS (K-Alpha, Thermo Fisher Scientific, Waltham, MA, USA). The gas sensing properties of the test materials, such as their sensitivity, operating temperature, T(res)/T(rec), selectivity, and repeatability, were mainly tested via an intelligent gas sensing analysis system (CGS-8, Beijing GIC-Tech Corp., Ltd., Beijing, China).

### 2.3. Preparation of ZnSnO_3_ and Bi-ZnSnO_3_

**Figure 1:**
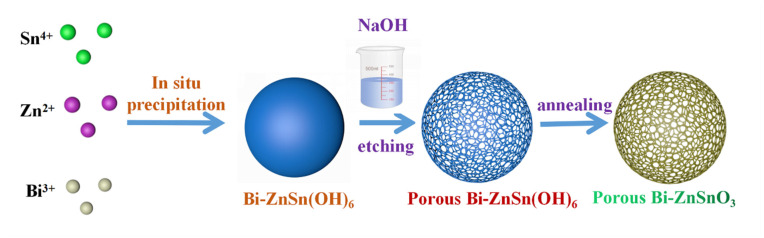
Synthesis process of Bi-ZnSnO_3_ composites.

ZnSnO_3_ was prepared through an in situ precipitation method. First, ethanol aqueous solution (33.3 wt%) was prepared. Next, 4 mmol SnCl_4_•5H_2_O and 4 mmol Zn(NO_3_)_2_•7H_2_O were dispersed into 64 mL of an ethanol aqueous solution, followed by magnetic stirring for 30 min to form the uniform suspension marked as solution A. Afterwards, 48 mmol NaOH was added to a beaker containing 48 mL of deionized water and completely dissolved, forming solution B, which was then slowly added into solution A to be subjected to magnetic stirring for 30 min. Then, 120 mmol NaOH was dissolved in 32 mL of ethanol aqueous solution to form a clear solution, which was added dropwise into the abovementioned mixed solution. After stirring for 5 min, Bi(NO_3_)_3_•6H_2_O with different mass fractions was added at the doping concentration of 0 wt%, 2 wt%, 4 wt%, 5 wt%, or 7 wt%, followed by magnetic stirring for 30 min. It was transferred to a 500 mL round-bottom flask for refluxing at 85 °C for 3 h and left to stand for 12 h. The precipitates were filtered, rinsed, and dried. The obtained powder materials were heated in a 450 °C tube furnace at a heating rate of 5 °C/min for 3 h, yielding Bi-ZnSnO_3_ with different Bi contents (pure ZnSnO_3_ was acquired at the Bi doping concentration of 0 wt%). The synthesis process of Bi-ZnSnO_3_ is displayed in Figure 1.

Under alkaline conditions, Zn^2+^, Sn^4+^, and Bi were doped to form the Bi-ZnSn(OH)_6_ precursor (Formula (1)). Then, Bi-ZnSn(OH)_6_ was calcinated at a high temperature, and crystals were removed to form Bi-ZnSnO_3_ (Formula (2)).

(1) $${\;\mathrm{Zn}}^{2+}+{\mathrm{Sn}}^{4+}+\mathrm{Bi}\to \mathrm{Bi}-\mathrm{ZnSn}\left(\mathrm{OH}\right){\;}_{6}$$

(2) $$\mathrm{Bi}-\mathrm{ZnSn}\left(\mathrm{OH}\right){\;}_{6}\to \mathrm{Bi}-{\mathrm{ZnSnO}}_{3}$$

### 2.4. Preparation of Sensor Device

First, a small amount of the ZnSnO_3_ and Bi-ZnSnO_3_ samples were weighed and uniformly ground in a mortar. Second, a moderate amount of absolute ethanol was added into the ground samples to dissolve them into pastes, which were uniformly coated on the outer surface of the gas sensor. Next, the sensor was placed in an 80 °C vacuum-drying oven for 12 h, followed by aging in a 200 °C aging system for 12 h. Finally, the gas-sensing properties of the aging sensor were explored with air-sensing test equipment. The sensitivity of gas sensitive materials was defined as follows:

(3) S=Ra/Rg

where Ra is the resistance (Ω) of the gas-sensing material in air and Rg is the resistance (Ω) of the gas-sensing material in the gas under test.

## 3. Results and Discussion

### 3.1. XRD

**Figure 2:**
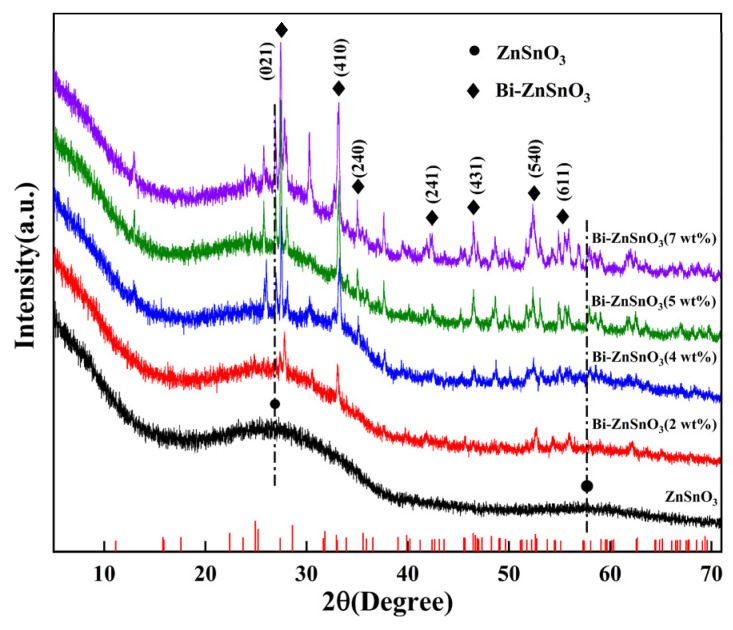
XRD patterns of ZnSnO_3_ and Bi-ZnSnO_3_.

The XRD patterns of ZnSnO_3_ and Bi-ZnSnO_3_ are exhibited in Figure 2. It was clearly observed that pure ZnSnO_3_ had two weak and wide diffraction peaks at 2θ = 27.25° and 58.59°, indicating that amorphous ZnSnO_3_ formed after the heat treatment [21]. This was because the calcination and dehydration of the precursor ZnSn(OH)_6_ destroyed the original H–O bond and changes the internal lattice arrangement, thereby forming amorphous ZnSnO_3_ [22]. In comparison with the patterns of pure ZnSnO_3_, new intense characteristic peaks could be observed in Bi-ZnSnO_3_ at 2θ = 27.47°, 33.16°, 35.01°, 42.42°, 46.47°, 52.38°, and 55.11°, which corresponded to the crystal faces of (021), (410), (240), (241), (431), (540), and (611), respectively. This result coincided with the standard data card (PDF#17-0320) of Bi, indicating that these newly generated diffraction peaks resulted from Bi doping. With the increase in the doping concentration of Bi, the intensity of diffraction peaks rose substantially, which further proved the successful doping of Bi.

### 3.2. SEM

**Figure 3:**
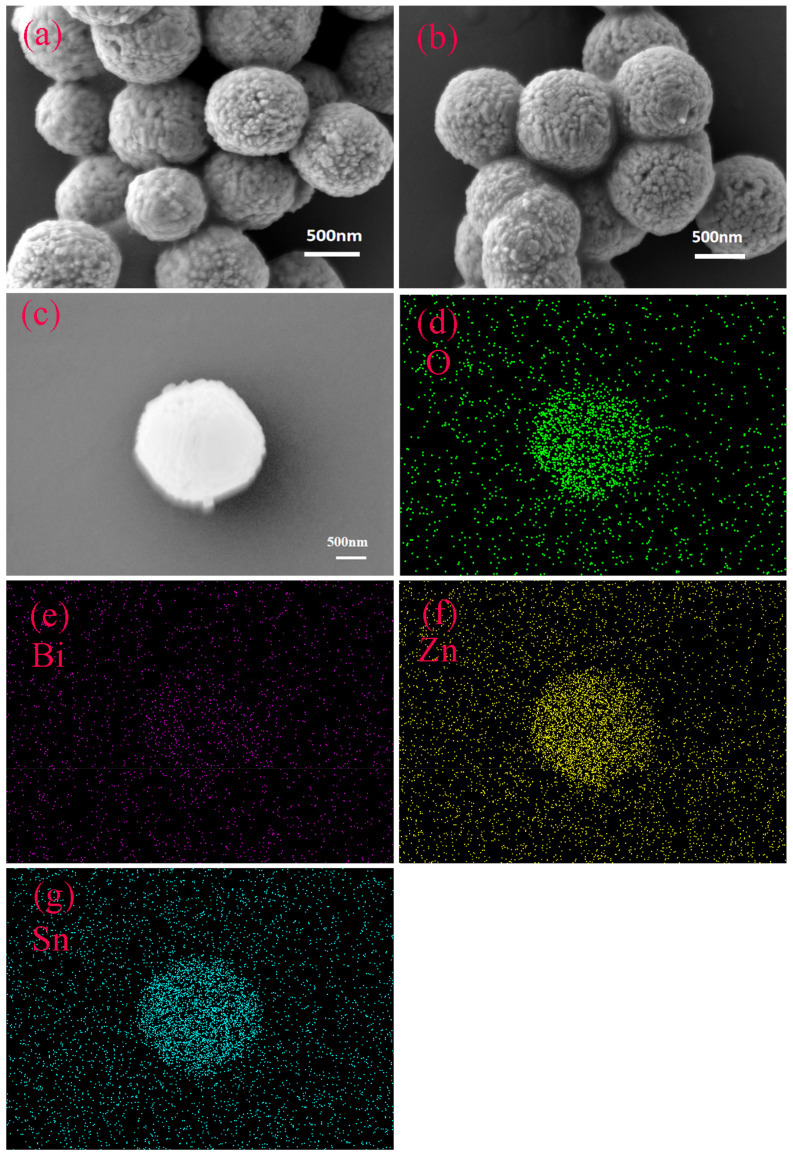
(**a**) SEM image of ZnSnO_3_, (**b**) Bi-ZnSnO_3_, and (**c**–**g**) elemental distribution of Bi-ZnSnO_3_.

The SEM images of pure ZnSnO_3_ and Bi-ZnSnO_3_ are presented in Figure 3. It can be seen from the SEM image (Figure 3a) of ZnSnO_3_ that pure ZnSnO_3_ had a spherical structure with a coarse appearance and many holes on the surface. From the gaps of broken holes, the pure ZnSnO_3_ was hollowly structured, with a large pore volume and a diameter of about 900 nm, and each ZnSnO_3_ block consisted of many tiny, uniformly distributed nanoparticles. As shown in Figure 3b, Bi-doped ZnSnO_3_ had a similar appearance, namely, it remained a spherical hollow porous structure. The element distribution diagram was mainly used to study the composition and concentration of each component in the test materials. As further verified by Figure 3c–g, four elements, O, Bi, Zn, and Sn, mainly existed in Bi-ZnSnO_3_, among which Sn, Zn, and O were distributed quite uniformly, though the element distribution does not seem obvious in the figures due to too small doping concentrations of Bi. The mass ratios and the atomic percentages of the four elements are listed in Table 1. It can be intuitively seen that the atomic percentages of O, Bi, Zn, and Sn were 47.54%, 3.43%, 20.44%, and 28.58%, respectively. Evidently, the content of Bi was much lower than that of the other three elements.

### 3.3. TEM

**Figure 4:**
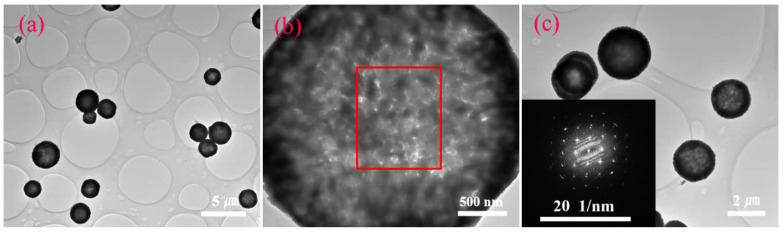
TEM image of pure ZnSnO_3_ (**a**,**b**) and TEM image of Bi-ZnSnO_3_ (4 wt%) (**c**); inset is the SAED image (**c**).

The microstructures of pure ZnSnO_3_ and Bi-ZnSnO_3_ were further explored via TEM. The TEM images of pure ZnSnO_3_ are displayed in Figure 4a,b. The spherically structured ZnSnO_3_ was 1.92 nm in size and was formed by the aggregation of many particles. In addition, the hollow structure of the sample clearly had many holes on it, in agreement with the SEM characterization results. As observed from the TEM image (Figure 4c) of Bi-ZnSnO_3_, the sample did change much morphologically, keeping a spherical hollow porous structure with a size of 1.98 nm. The electronic diffraction pattern is illustrated in Figure 4c, presenting regularly arranged diffraction spots. Given this, Bi-ZnSnO_3_ was judged to be a single-crystal structure within the range of selection.

### 3.4. BET

**Figure 5:**
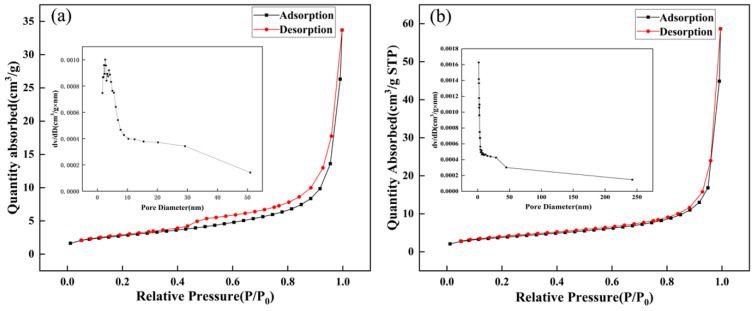
Nitrogen adsorption–desorption isotherms of ZnSnO_3_ (**a**) and Bi-ZnSnO_3_ (4 wt%) composites (**b**); the inset is the BJH pore size distribution curve.

Specific surface area is one of the important parameters for measuring gas-sensitive materials. In this study, the specific surface area of prepared materials was determined through the N_2_ adsorption–desorption method. The N_2_ adsorption–desorption isothermal diagram of pure ZnSnO_3_ and Bi-ZnSnO_3_ is displayed in Figure 5a,b. According to the classification of International Union of Pure and Applied Chemistry (IUPAC), both materials had a type IV isothermal forming an H3 hysteresis loop, which is the characteristic isothermal of mesoporous materials [23,24]. The BET specific surface areas of pure ZnSnO_3_ and Bi-ZnSnO_3_ were 10.01 m^2^/g and 13.75 m^2^/g, respectively. The pore volume and the average pore size of pure ZnSnO_3_ were 0.02 cm^3^/g and 9.26 nm, and those of Bi-ZnSnO_3_ were 0.03 cm^3^/g and 25.51 nm. The pores might have been generated by the gaps generated in the formation of ZnSnO_3_ nanospheres. Furthermore, the mesoporous structure of microspheres and the enlargement of their specific surface area facilitated the gas adsorption and further enhanced the gas transport efficiency [25]. Therefore, we hypothesize that the changes in the specific surface area, the pore volume, and the pore size of Bi-ZnSnO_3_ exerted important influences on the gas sensitivity of this material.

### 3.5. XPS

**Figure 6:**
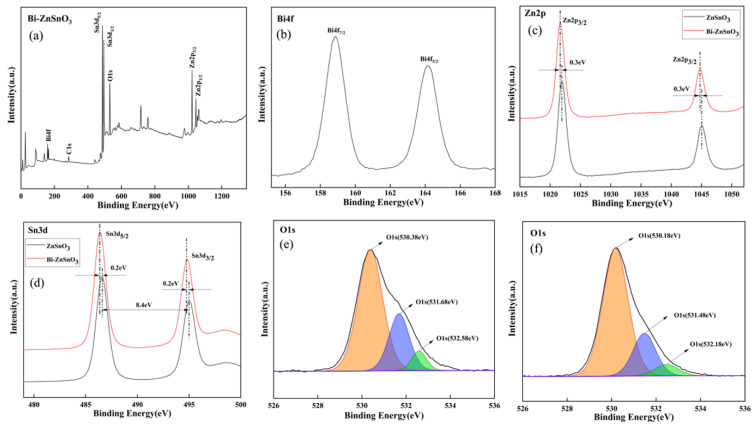
XPS full spectrum of Bi-ZnSnO_3_ (4 wt%) composite (**a**), high-resolution spectrum of Bi4f (**b**), high-resolution spectrum of Zn2p (**c**), high-resolution spectrum of Sn3d (**d**), and O1s spectra of ZnSnO_3_ and Bi-ZnSnO_3_ (4 wt%) composites (**e**,**f**).

The surface chemical composition and element distribution were analyzed via XPS. The XPS survey spectrum of Bi-ZnSnO_3_ is displayed in Figure 6a. Characteristic peaks of C, Zn, Sn, O, and Bi existed in the composites, indicating the presence of these five elements in the samples. The high-resolution pattern of Bi4f is exhibited in Figure 6b, where the peaks at the binding energy of 159.08 eV and 164.08 eV corresponded to Bi4f_7/2_ and Bi4f_5/2_ of Bi, respectively. The high-resolution patterns of Zn2p in pure ZnSnO_3_ and Bi-ZnSnO_3_ are shown in Figure 6c. The binding energies of Zn2p_3/2_ and Zn2p_1/2_ in Bi-ZnSnO_3_ were 1021.68 and 1044.78 eV, respectively, which showed a shift of 0.3 eV in comparison with the binding energy (1021.98 eV and 1045.08 eV) in pure ZnSnO_3_. This may be because Bi doping changed the electron density on the ZnSnO_3_ surface and reduced the binding energy. The high-resolution patterns of Sn3d in pure ZnSnO_3_ and Bi-ZnSnO_3_ are displayed in Figure 6d. The binding energies of Sn3d_5/2_ and Sn3d_3/2_ in Bi-ZnSnO_3_ were located at 486.38 and 494.78 eV, respectively, showing a shift of 0.2 eV compared with the binding energy (486.58 eV and 494.98 eV) in pure ZnSnO_3_, another change that we attributed to Bi doping. In addition, the energy gap between two corresponding peaks of Sn3d was 8.4 eV, indicating that Sn existed in the form of +4 valence [26]. The high-resolution patterns of O1s in pure ZnSnO_3_ and Bi-ZnSnO_3_ are displayed in Figure 6e,f, where the O1s peak could be divided into three characteristic peaks: lattice oxygen, oxygen vacancies, and chemi-adsorbed oxygen. From Figure 6e, the three characteristic peaks of pure ZnSnO_3_ were successively located at 530.18, 531.48, and 532.48 eV, respectively, with area ratios of 72.28%, 21.68%, and 6.04%, respectively. As shown in Figure 6f, the three characteristic peaks of Bi-ZnSnO_3_ were located at 530.38, 531.68, and 532.58 eV, with area ratios of 67.34%, 26.10%, and 6.56%, respectively. It is widely accepted that the higher the proportions of oxygen vacancies and chemi-adsorbed oxygen are, the better the gas sensing properties of the sensor will be [27,28]. In this study, the total proportion of oxygen vacancies and chemi-adsorbed oxygen in Bi-ZnSnO_3_ was 32.66%, which was higher than 27.72% in pure ZnSnO_3_, further proving that Bi replaced the lattice atoms in pure ZnSnO_3_ so as to form oxygen defects.

### 3.6. The Working Temperature of Bi-ZnSnO_3_

**Figure 7:**
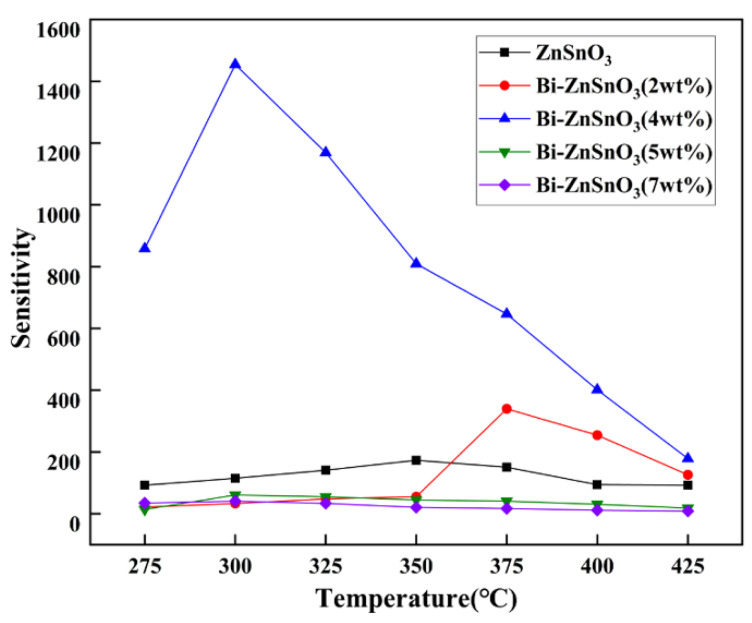
Temperature response curves of pure ZnSnO_3_ and different ratios of Bi-ZnSnO_3_ to 100 ppm n-butanol gas.

In general, the properties of metal-oxide gas sensors are closely related to their operating temperature [29]. Too high of an operating temperature not only increases the wear rate of gas-sensitive elements but also raises their application and production costs, so a significant research objective in the field of metal-oxide gas sensors is to reduce their operating temperature [30]. Figure 7 shows the temperature response curves of pure ZnSnO_3_ and Bi-ZnSnO_3_ of different mass ratios to 100 ppm *n*-butanol gas. Under normal circumstances, the sensitivity of gas sensors would first grow and then decline with the rise in ambient temperature. This is because both the adsorption rate and the desorption rate are accelerated by a rise in the ambient temperature, but the former is faster than the latter, thus substantially enhancing the material sensitivity. As the ambient temperature further rises after reaching a certain level, the acceleration of the desorption rate exceeds that of the adsorption rate, thus lowering the sensitivity of the gas-sensitive material [31,32]. First, the temperature response of Bi-ZnSnO_3_ at the doping concentrations of 0 wt%, 2 wt%, 4 wt%, 5 wt%, and 7 wt% was tested. It can be clearly observed from the temperature response diagram that the five composites operated optimally at 350 °C, 375 °C, 300 °C, 300 °C, and 300 °C, corresponding to the respective sensitivities of 173.25, 339.56, 1450.65, 60.71, and 40.81. Thus, the Bi-ZnSnO_3_ reached the highest sensitivity at the doping concentration of 4 wt%, being 8.37 times that of pure ZnSnO_3_. Moreover, the optimal operating temperature of the composite was 300 °C, 50 °C lower than that of pure ZnSnO_3_. The above results show that the optimal proportion of Bi is doped in ZnSnO_3_ can effectively improve the gas-sensing properties of the matrix material ZnSnO_3_ in addition to lowering the optimal operating temperature.

### 3.7. The Sensitivity Performance of Bi-ZnSnO_3_ for N-Butanol

**Figure 8:**
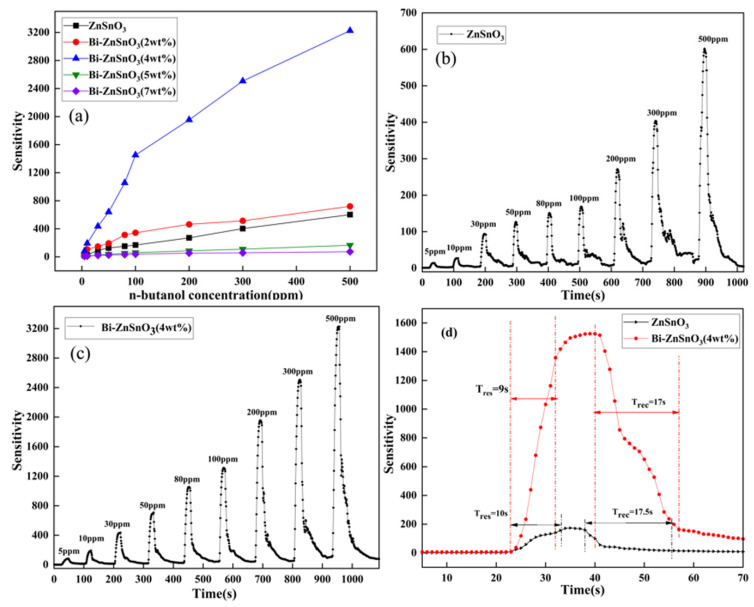
The relationship between the sensitivity of different materials and the concentration of n-butanol gas (**a**), the concentration–response curve of pure ZnSnO_3_ material to n-butanol gas at 325 °C (**b**), the concentration–response curve of Bi-ZnSnO_3_ to n-butanol gas at 300 °C (**c**), and the response recovery curve of pure ZnSnO_3_ and Bi-ZnSnO_3_ (4 wt%) materials to 100 ppm n-butanol gas (**d**).

As shown in Figure 8a, the sensitivities of ZnSnO_3_ and Bi-ZnSnO_3_ with different contents of Bi increased to various extents as the concentration of *n*-butanol gas increased from 5 to 500 ppm, with Bi-ZnSnO_3_ (4 wt%) presenting the fastest growth rate in sensitivity, which indicated that the optimal doping concentration of Bi was 4 wt%. In addition, the growth rate of Bi-ZnSnO_3_ (4 wt%) first increased and then declined with the increase in the concentration of *n*-butanol gas. This was because a rise in the concentration of *n*-butanol brings about an increasing number of molecules that occupy a limited number of adsorption sites on the surface of the sensing material, which eventually become saturated and, thus, slow the growth in sensitivity. The concentration–response curves of pure ZnSnO_3_ and Bi-ZnSnO_3_ (4 wt%) to *n*-butanol gas are shown in Figure 8b,c, respectively. The sensitivity of pure ZnSnO_3_ to various concentrations of *n*-butanol gas was 14.01, 27.02, 93.82, 126.89, 151.29, 168.67, 271.28, 403.45, and 601.87, while that of Bi-ZnSnO_3_ to *n*-butanol gas at various concentrations was 82.76, 192.36, 435.06, 693.65, 1055.08, 1450.65, 1953.68, 2506.184, and 3226.02. It was clear that the sensitivity of the two gas-sensitive materials to *n*-butanol gas increased progressively with the *n*-butanol gas concentration. That is because more and more available *n*-butanol molecules participate in the redox reaction, contributing to the gradual increase in the sensitivity of the materials. At a given *n*-butanol gas concentration, the sensitivity of Bi-ZnSnO_3_ (4 wt%) was much higher than that of pure ZnSnO_3_ due to the modification of Bi. Additionally, the repeatability of the gas sensor was excellent, as the sensitivity increased rapidly after adsorbing *n*-butanol gas and quickly returned to the initial state when it was exposed to the air.

T(res)/T(rec) plays an important role in the evaluation of the properties of sensors [33,34]. T(res) is defined as the time it takes for a gas sensor to reach 90% of its maximum sensitivity to the measured gas, while T(rec) stands for the time it takes for the resistance of a gas sensor to reach 90% of its resistance variation when the sensor is away from the measured gas. We drew the response curves of the two materials to 100 ppm *n*-butanol gas at their respective optimal operating temperatures (Figure 8d). The T(res) and T(rec) of pure ZnSnO_3_ were 10 s and 17.5 s, while those of Bi-ZnSnO_3_ were 8 s and 17 s. By comparison, the T(res) and T(rec) of Bi-ZnSnO_3_ (4 wt%) were significantly shorter than those of ZnSnO_3_, meaning that the response and recovery performance of the composites was significantly higher, which in turn improved the application value of the materials.

**Figure 9:**
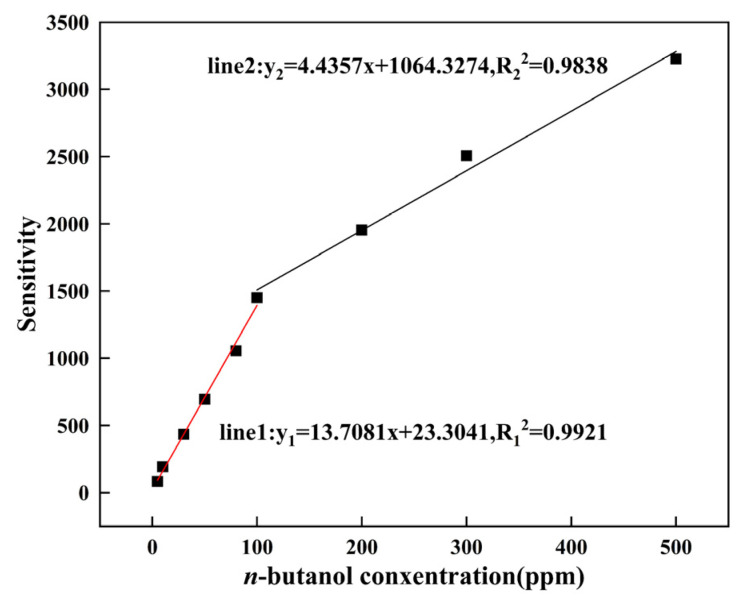
Linear relationship between material sensitivity and *n*-butanol concentration of Bi-ZnSnO_3_ (4 wt%).

The linear relationship between the sensitivity of Bi-ZnSnO_3_ and the concentration of *n*-butanol gas (5–500 ppm) is shown in Figure 9, from which it can be seen that, with the increase in gas concentration, the sensitivity of Bi-ZnSnO_3_ (4 wt%) composites increased rapidly in the *n*-butanol environment with a concentration of less than 100 ppm. When the *n*-butanol concentration exceeded 100 ppm, the sensitivity of the composites increased rapidly. This indicates that the detection limit of the material gradually approaches a peak, so it may be necessary to modify the material in other ways to better detect higher concentrations of *n*-butanol gas. The two-stage linear correlation coefficients of the Bi-ZnSnO_3_ (4 wt%) composite are R_1_^2^ = 0.9921and R_2_^2^ = 0.9838, respectively. The results show that the composite can accurately detect n-butanol gas in the range of 5 ppm–500 ppm in practical application. 

### 3.8. Sensitivity of ZnSnO_3_ and Bi-ZnSnO_3_ to Different VOCs

**Figure 10:**
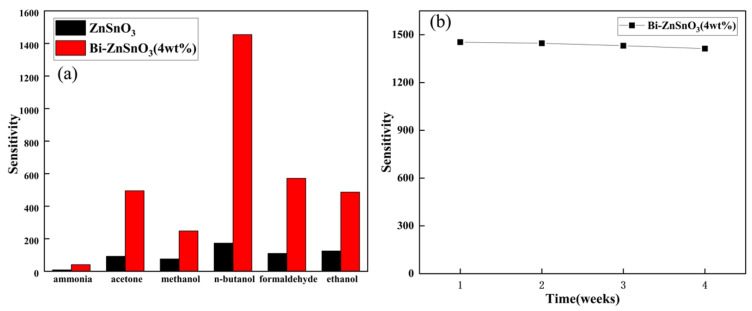
(**a**) Comparison of selectivity test results of pure ZnSnO_3_ and Bi-ZnSnO_3_ (4 wt%) and (**b**) repeatability test results of Bi-ZnSnO_3_ (4 wt%).

Its good selectivity can be attributed to the different adsorption capacities and reducibilities of the sensor surface for various measured gases. To further evaluate the selectivity of the two sensor materials, the sensitivities of pure ZnSnO_3_ and Bi-ZnSnO_3_ (4 wt%) gas-sensitive elements to six different volatile organic compounds at 100 ppm were measured at their optimal operating temperatures. The results are shown in Figure 10a. The sensitivity of Bi-ZnSnO_3_ (4 wt%) to *n*-butanol gas reached 1450.65, which was 35.37 times that (41.01) of ammonia gas, 2.93 times that (495.09) of acetone gas, 6.02 times that (241.05) of methanol gas, 2.54 times that (571.48) of formaldehyde gas, and 2.98 times that (486.58) of ethanol gas. By comparison, the sensitivity of Bi-ZnSnO_3_ (4 wt%) to *n*-butanol was much higher than that of other gases, demonstrating a great improvement over pure ZnSnO_3_. Additionally, Bi-ZnSnO_3_ presented excellent selectivity for *n*-butanol gas, probably because Bi doping increased the specific surface area of ZnSnO_3_ and gave rise to many active sites on the surfaces, thus improving its gas-sensing properties.

The response values of Bi-ZnSnO_3_ (4 wt%) to a gas concentration of 100 ppm at 300 °C were tested repeatedly within 28 days (four times, once a week) (Figure 10b). The sensitivity obtained from the first test was 1450.65, while that of the fourth test was 1431.60, which indicated that the sensitivity of the composites decreased slightly as the test progressed. Even so, the sensitivity remained above 95% of the initial response value, showing that this gas-sensitive material has good stability for repeated use.

The performance parameters of the Bi-ZnSnO_3_ gas sensor to *n*-butanol are compared with those of other gas sensors in Table 2. Despite a higher operating temperature, the Bi-ZnSnO_3_ gas sensor showed a much better sensitivity to *n*-butanol gas at 100 ppm than other gas sensors based on different gas-sensitive materials. The optimal operating temperature of the ZnSnO_3_ sensor was reduced to 300 °C due to the doping of Bi. All these findings indicate that Bi-ZnSnO_3_, serving as coating materials for gas sensors, is significantly valuable in research and in real-world applications.

### 3.9. The Sensing Mechanism

The adsorption and desorption of oxygen molecules on the surfaces of n-type semiconductors lead to resistance changes, so the gas-sensing properties of n-type semiconductors were compared [41]. When ZnSnO_3_ is exposed to air, oxygen molecules in the air will be adsorbed on its surface to form chemi-adsorbed oxygen, generating ionized oxygen $O_{2}^{-},$ $O^{-},$ and $O^{2-}$. As the temperature increases, oxygen molecules capture free electrons from the conduction band of ZnSnO_3_, resulting in a decrease in electron concentration and the formation of a depletion layer, which increases the resistance of the gas sensor [42]. Hence, the sensitive mechanism of n-butanol detection can be illustrated by the Wolkenstein model [43], as follows:

(4) $$O_{2}\left(\mathrm{gas}\right)\to O_{2}\left(\mathrm{ads}\right)$$

(5) $$O_{2}\left(\mathrm{ads}\right){+e}^{-}\to O_{2}^{-}\left(\mathrm{ads}\right)$$

(6) $$O_{2}^{-}\left(\mathrm{ads}\right){+e}^{-}\to 2O^{-}\left(\mathrm{ads}\right)$$

(7) $$O^{-}\left(\mathrm{ads}\right){+e}^{-}\to O^{2-}\left(\mathrm{ads}\right)$$

The reaction in Formulas (4) and (5) mainly occurred at a temperature less than 100 °C (T < 100 °C); the reaction in Formula (6) mainly occurred when 100 °C < T < 300 °C; and the reaction in Formula (7) mainly occurred when T > 300 °C. A redox reaction between *n*-butanol gas and ionized oxygen species is initiated by the contact of gas-sensitive elements and *n*-butanol gas, which releases the captured electrons back to the conduction band of ZnSnO_3_, reducing the thickness of the electron depletion layer, increasing the carrier concentration, and lowering the resistance of the gas-sensitive elements. According to the test results, the optimal operating temperatures of the prepared materials are above 300 °C, and the chemical reactions in this process are mainly as follows [44,45,46]:

(8) $$C_{4}H_{9}\mathrm{OH}+12O^{-}\left(\mathrm{ads}\right)\to {4\mathrm{CO}}_{2}+5H_{2}O+12e^{-}$$

(9) $$C_{4}H_{9}\mathrm{OH}+12O^{2-}\left(\mathrm{ads}\right)\to {4\mathrm{CO}}_{2}+5H_{2}O+24e^{-}$$

**Figure 11:**
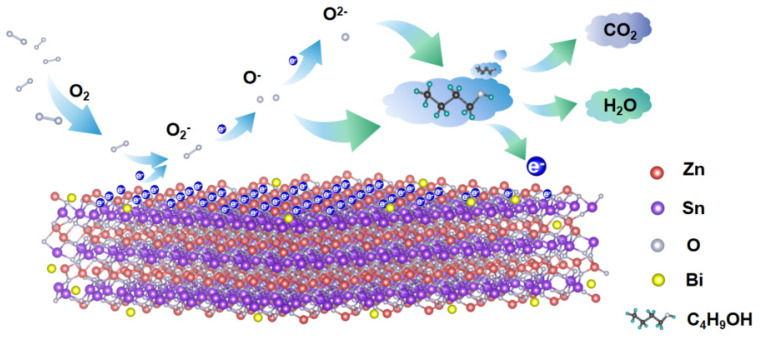
Gas−sensing mechanism of Bi-ZnSnO_3_ composites.

Combined with the XPS results, these results show that the total proportion of oxygen vacancies and chemi-adsorbed oxygen in Bi-ZnSnO_3_ (4 wt%) was from 27.72% to 32.68% higher than that of pure ZnSnO_3_. The increase in oxygen vacancies indicated that more electrons could be captured from the conduction band of Bi-ZnSnO_3_ while chemi-adsorbed oxygen directly participated in the reaction of *n*-butanol gas to release more electrons back to the conduction band of Bi-ZnSnO_3_, leading to a stronger change in the electron depletion layer. This suggests that the introduction of Bi is beneficial to the surface adsorption of oxygen. In addition, according to the BET characterization, the specific surface area of Bi-ZnSnO_3_ was also improved, which led to more active sites on the gas sensor surface. Finally, the gas-sensing properties of Bi-ZnSnO_3_ were promoted by a more complete electron depletion layer due to an increasing number of oxygen molecules adsorbed on the surface [47]. The gas-sensing mechanism of Bi-ZnSnO_3_ is shown in Figure 11.

## 4. Conclusions

ZnSnO_3_ and Bi-ZnSnO_3_ were first synthesized via the in situ precipitation method and then were characterized as nanospheres, followed by a study on the gas-sensing properties to n-butanol gas. The test results revealed that Bi-ZnSnO_3_, compared with pure ZnSnO_3_, was a superior sensor of n-butanol gas. In particular, Bi-ZnSnO_3_ (4 wt%) possessed the highest sensitivity of 1450.65, approximately 8.37 times that of pure ZnSnO_3_, at an optimal operating temperature of 300 °C, which was 50 °C lower than that of pure ZnSnO_3_. Moreover, Bi-ZnSnO_3_ had better selectivity and repeatability. The total proportion of oxygen vacancies and chemi-adsorbed oxygen in Bi-ZnSnO_3_ (4 wt%) was from 27.72% to 32.68% higher than that of pure ZnSnO_3_. The increase in oxygen vacancies indicated that more electrons could be captured from the conduction band of Bi-ZnSnO_3_, while chemi-adsorbed oxygen directly participated in the reaction of n-butanol gas to release more electrons back to the conduction band of Bi-ZnSnO_3_, leading to a stronger change in the electron depletion layer. Altogether, our results suggest that Bi-ZnSnO_3_ have great potential in the detection of n-butanol gas owing to its excellent gas-sensing properties, in contrast to traditional n-butanol sensors.

## Figures and Tables

**Table 1:** Elemental mass ratio and atomic percentage of Bi-ZnSnO_3_ (4 wt%).

Element	Weight%	Atomic%
O	13.18	47.54
Bi	12.43	3.43
Zn	42.03	20.44
Sn	32.37	28.58

**Table 2:** Performance comparison of *n*-butanol gas sensor.

Material	Working Temperature (°C)	Gas Concentration (ppm)	Response Time/Recovery Time (s)	Sensitivity	Reference
ZnO	340	100	6/17	136.00	[35]
CdIn_2_O_4_	2800	100	4/10	81.20	[36]
α-Fe_2_O_3_	280	100	5/5	13.90	[37]
In-TiO_2_/WO_3_	200	50	2.2/3	127.00	[38]
PtO_2_/CuO	180	100	2.4/5.1	11.55	[39]
In_2_O_3_	140	100	45/65	241.00	[40]
Bi-ZnSnO_3_	300	100	8/17	1450.65	this paper
